# Cost-effectiveness of implant-supported dental prosthesis compared to conventional dental prosthesis

**DOI:** 10.11606/s1518-8787.2019053001066

**Published:** 2019-08-15

**Authors:** Livia Fernandes Probst, Tazio Vanni, Denise de Fátima Barros Cavalcante, Erica Tatiane da Silva, Yuri Wanderley Cavalcanti, Luis Augusto Passeri, Antonio Carlos Pereira

**Affiliations:** I Universidade Estadual de Campinas. Faculdade de Odontologia de Piracicaba. Programa de Pós-Graduação em Odontologia. Piracicaba, SP, Brasil; II MBA em Economia e Avaliação de Tecnologias em Saúde. Hospital Alemão Oswaldo Cruz. São Paulo, SP, Brasil; III Instituto Butantan. Divisão de Ensaios Clínicos e Farmacovigilância. São Paulo, SP, Brasil; IV Fiocruz Brasília. Programa de Evidências para Políticas e Tecnologias em Saúde. Brasília, DF, Brasil; V Universidade Federal da Paraíba. Departamento de Clínica e Odontologia Social. João Pessoa, PB, Brasil; VI Universidade Estadual de Campinas. Faculdade de Ciências Médicas. Departamento de Cirurgia. Campinas, SP, Brasil; VII Universidade Estadual de Campinas. Faculdade de Odontologia de Piracicaba. Departamento de Odontologia Social. Piracicaba, SP, Brasil

**Keywords:** Jaw, Edentulous, rehabilitation, Dental Prosthesis, economics, Cost-Effectiveness Evaluation, Unified Health System

## Abstract

**OBJECTIVE:**

To conduct a cost-effectiveness analysis of alternatives for rehabilitation treatment of mandibular edentulism in the context of the Brazilian Unified Health System (implant-supported total dental prosthesis *versus* conventional total dental prosthesis).

**METHODS:**

A Markov model was developed to capture long-term clinical and economic outcomes. The model’s population was comprised of a hypothetical cohort of 1,000,000 patients, aged 55 years, with total mandibular edentulism and without medical contraindications for performing surgical procedures. The adopted analysis perspective was that of the Brazilian Unified Health System. Based on the proposed model, we calculated cost – in BRL, and effectiveness – measured by quality-adjusted prosthesis year (QAPY). The time horizon of the analysis was 20 years.

**RESULTS:**

Considering a 5% discount in costs and effects, the incremental cost-effectiveness ratio of implant-supported total dental prostheses compared to conventional total dental prosthesis (BRL 464.22/QAPY) was lower than the willingness to pay threshold adopted in the model (BRL 3,050.00/QAPY).

**CONCLUSIONS:**

The results of this economic analysis showed that the rehabilitation of mandibular edentulous patients by implant-supported total prosthesis is very cost-effective when compared to conventional complete prosthesis, considering the cost-effectiveness limits employed.

## INTRODUCTION

Untreated edentulism is a serious public health problem, affecting approximately 276 million people worldwide. It is the main cause of disability due to oral conditions, as estimated by disability-adjusted life years (DALY)^1^. Edentulism is defined as an oral health condition stemming from the failure or lack of timely application of all preventive possibilities^2^.

Despite the advances achieved by Brazil’s National Oral Health Policy^3^, the prevalence of this condition remains high. The National Health Survey conducted in 2013 showed that a total of 16 million Brazilians had total tooth loss^4^. Mandibular edentulism affects an even higher number of people: 31.23% adults, and 67.29% older adults over 60 years old. The reality is no different in the state of São Paulo. Although it is the richest in the country, its older adults have a high average of extracted teeth (25.87%); often requiring mandibular rehabilitation treatment (37.27%)^5^. Such information is highly relevant due to pointing out the importance of public policies for the treatment of oral diseases.

Patients with extensive dental loss who do not undergo prosthetic rehabilitation have their quality of life reduced^6^. This is mainly due to the effects of difficulties with chewing and eating^7^over their well-being, appearance and social life^2,6^. In this context, oral rehabilitation by prostheses can have a positive effect, as it adequately restores masticatory function and aesthetics, contributing to improvements in social interaction and quality of life^8^.

When it comes to the rehabilitation of patients with complete edentulism, rehabilitation with implant-supported total prosthesis (ISTP) offers greater quality of life benefits than conventional total prosthesis (CTP)^9^. However, although conventional total prostheses are generally less functional and have limited comfort, aesthetics, and occlusal stability, their use remains a viable and very frequent treatment option in dental clinics, especially for budget-restricted social strata^10^.

The federal government finances two oral rehabilitation alternatives for edentulous patients: CTP^11^ and ISTP^12^. However, dental specialty centers in Brazil are yet to effectively adhere to the provision of implant rehabilitation. Data from the first cycle of the Program for the Improvement of Access and Quality (PMAQ-CEO), held in 2014, show that only 1.8% of Brazil’s dental specialty centers have professionals working in the area of implantology. This demonstrates that, existing regulations for financing dental implants notwithstanding, additional measures need to be adopted in order to boost the supply of implant prostheses. Recently, the Ministry of Health (MS) stipulated that specialty centers that still need to implement the service should now put it into effect after a technical and budgetary analysis^13^. The latter denotes the ministerial concern with financial resource allocation.

The high cost of oral health care services is recognized worldwide, and is especially problematic where public assistance programs at different levels of care are concerned^14,15^. It is estimated that, in 2015, the global spending on dental treatments reached the sum of USD 356.80 billion, not to mention USD 187.61 billion in productivity losses caused by oral problems^16^. Considering how limited financial resources are, it is important for public health systems to use them as effectively as possible. In this sense, economic evaluation studies can help ensure efficiency, especially when indicating ways to prioritize assistance in the use the available resources^14^. However, no cost-effectiveness studies comparing these two implant technologies from the perspective of the Brazilian Unified Health System (SUS) were found.

Considering the relevance of the subject of edentulism in the public health sphere, as well as its high prevalence in the Brazilian population, this study evaluates the cost-effectiveness of ISTP *versus* CTP in rehabilitation of mandibular edentulism, specifically in the context of older adults serviced by the SUS.

## METHODS

### Research Question

What is the incremental cost-effectiveness ratio of implant-supported total prosthesis instead of conventional total prosthesis for mandibular edentulous rehabilitation, considering a time horizon of 20 years and the Ministry of Health’s perspective?

### Study Design

A complete economic evaluation of cost-effectiveness based on mathematical modeling and built in accordance with the Economic Evaluation Guidelines of the Brazilian Network for Health Technology Assessment (Rebrats)^17^.

### Perspective

This analysis adopted the perspective of the Ministry of Health, responsible for managing the SUS at a federal level.

### Target Population

The model’s population was comprised of a hypothetical cohort of 1,000,000 patients, aged 55 years, with total mandibular edentulism and without medical contraindications for performing surgical procedures.

### Interventions

The intervention of interest was the ISTP prosthetic rehabilitation treatment of mandibular edentulous older adults. This rehabilitation can employ different techniques, but in our model only the mandibular two-implant supported overdenture was considered, since the scientific evidence points to it as the minimum treatment indicated^18^ for the rehabilitation of edentulous mandibles.

CTP was chosen as a comparator since it is the most widespread rehabilitation technique in the SUS, and continues to be routinely used in dental practice, due to its low cost^10^.

The number of consultations required and the sequential procedures of each intervention were modelled according to the traditional protocol of techniques. Primary Care Book nº 17^21^, which deals with issues concerning the referral of primary care SUS patients to secondary care, was also employed as a reference. These issues were considered because, although know-how on implementation of prostheses is not mandatory in specialty centers, municipalities usually have partnerships with these centers, and use them to absorb excess demand, hiring specialists who work according to production quotas agreed upon by both parties.

### Discount Rate and Time Horizon

An annual discount rate of 5% for both costs and effectiveness was applied, following Rebrats guidelines^17^. Considering the Brazilian average life expectancy^22^ of 75.8 years, and the durability of the assessed technologies, the time horizon of the analysis was defined as 20 years.

### Model Structure

**Figure 1:**
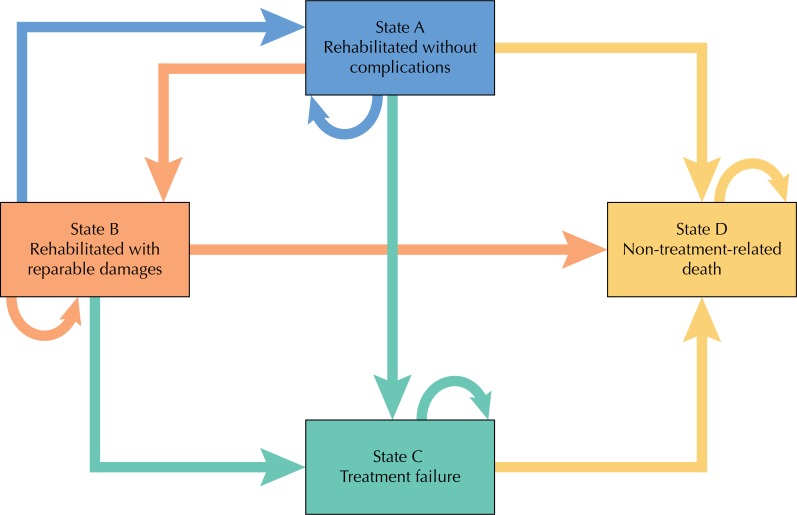
Model structure. Rehabilitation can employ conventional prosthesis or implant-supported total prosthesis.

We developed a Markov model to capture the long-term clinical and economic results of implant-supported total prostheses as compared to conventional total prostheses (Figure 1). The model consists of four mutually exclusive health states: rehabilitated without complications (state A), rehabilitated with reparable damages (state B), treatment failure (state C), and non-treatment-related death (state D). Non-treatment-related death was included in the model due to the advanced age of the hypothetical cohort patients.

State B (repairable damages) contains:

- in CTP: soft tissue complications, artificial tooth loss, need for implant reinsertion due to loss of retention and stability;

- in ISTP: loss of the prosthetic connector, loss of artificial tooth, need for realignment.

State C (treatment failure) contains:

- in CTP: catastrophic fracture of the prosthesis, abandonment of the prosthesis;

- in ISTP: implant loss.

CTP: conventional total prosthesis; ISTP: implant-supported total prosthesis

The model predicts that patients may have transitioned between health states or remained in the same state at the end of the annual cycle. These transitions take place according to preassigned probabilities. Patients in state C are able to remain in that state or transition to death. Arrows indicate paths. The Markov model for each treatment was performed independently.

### Input Model Parameters

#### Effectiveness measure

There are still few cost-effectiveness evaluations of dental interventions. It is worth pointing out that, compared to medical interventions, dentistry interventions impact effectiveness in a different way. To cite an example, dentistry interventions generally have no direct impact on mortality. Thus, the quality-adjusted life year (QALY), commonly used in medical studies’ cost-utility analyses, does not have sufficient specificity to evaluate dentistry interventions.

The quality-adjusted prosthetic year (QAPY) is a measure derived from the QALY. Its values range from 0 (absent tooth) to 1 (prosthesis in perfect condition after one year)^23^. Since it provides a more adequate representation of the clinical results of the treatments evaluated here, we chose QAPY as the measure of outcome to be used in estimating effectiveness.

The QAPY values defined in this study were obtained from the calculation proposed by Chun et al.^24^, which considers patients’ degree of satisfaction according to aesthetics, function and phonation. Satisfaction values were obtained from a study by Farias-Neto et al.^25^, who investigated satisfaction in relation to both treatments in a sample of Brazilian patients. The study used the same subdivisions (dissatisfied, satisfied, and very satisfied) to rate aesthetic, functional and phonation criteria, so we were able to input its results into the formula by Chun et al.^24^ A sensitivity analysis was performed in order to investigate variation. QAPY values were 0.79 for patients who received CTP, 0.94 for patients who received ISTP, and zero for fully edentulous patients.

#### Costs

For the calculation of costs, the following parameters were used:

1. Only direct federal government costs were included, since the cost-effectiveness analysis adopted the perspective of the Ministry of Health.
2. Cost data were collected based on the top-down or macro-cost approach, and obtained from the SUS system known as the *Sistema de Gerenciamento da Tabela de Procedimentos, Medicamentos e OPM*^a^ (SIGTAP – Management System for the Table of Procedures, Medications and OPM)^26^.

Cost data for each modeled state are shown in Table 2 and expressed in BRL (year of 2018). CTP had an initial cost of BRL 196.83, compared to BRL 1,050.40 for two-implant ISTP.

**Table 2:** Estimated intervention costs (in BRL), used in the reference scenario of the budget impact analysis.

Costs of the conventional total prosthesis					
Procedure	Source	Accrual month	Value (BRL)	Quantity	Total
Panoramic radiograph	Sigtap^26^	March/2018	9.03	1	9.03
Consultation of higher education professionals in primary care (except doctor)	Sigtap^26^	March/2018	0	1	0
Consultation of higher education professionals in specialized care (except doctor)	Sigtap^26^	March/2018	6.30	6	37.80
Occlusal adjustment	Sigtap^26^	March/2018	0	1	0
Complete mandibular prosthesis	Sigtap^26^	March/2018	150.00	1	150.00

Total					196.83

**Costs of the implant-supported total prosthesis**					

Procedure	Source	Accrual month	Value (BRL)	Quantity	Total
Computed tomography of the face/sinuses/temporomandibular joints	Sigtap^26^	March/2018	173.50	1	173.50
Consultation of higher education professionals in primary care (except doctor)	Sigtap^26^	March/2018	0	1	0
Consultation of higher education professionals in specialized care (except doctor)	Sigtap^26^	March/2018	6.30	9	56.70
Osseointegrated dental implants	Sigtap^26^	March/2018	260.10	2	520.20
Dental prosthesis on implant	Sigtap^26^	March/2018	300.0	1	300
Occlusal adjustment	Sigtap^26^	March/2018	0	1	0

Total					1,050.40

SIGTAP: *Sistema de Gerenciamento da Tabela de Procedimentos, Medicamentos e OPM* (Management System for Table of Procedures, Medications and OPM)

#### Probabilities

Probabilities of transition between the model’s four different states are shown in Table 3.

**Table 1:** Model’s transition probabilities.

Parameters	Price	Reference
Transition probabilities in CTP		
State A to state A	0.1697	^*^
State A to state B	0.5121	^27^
State A to state C	0.3106	^27^
State A to state D	0.0075	^12^
State B to state A	0.5403	^28^
State B to state B	0.2231	^28^
State B to state C	0.2291	^28^
State B to state D	0.0075	^22^
State C to state C	0.9925	^*^
State C to state D	0.0075	^22^
State D to state D	1.0000	Death state
Transition probabilities in ISTP		
State A to state A	0.9154	^24^
State A to state B	0.0768	^24^
State A to state C	0.0003	^24^
State A to state D	0.0075	^22^
State B to state A	0.6519	^28^
State B to state B	0.2909	^*^
State B to state C	0.0496	^28^
State B to state D	0.0075	^22^
State C to state C	0.9925	^*^
State C to state D	0.0075	^22^
State D to state D	1.0000	Death state

CTP: conventional total prosthesis; ISTP: implant-supported total prosthesis; state A: rehabilitated without complications; state B: rehabilitated with reparable damages; state C: treatment failure; state D: non-treatment-related death

* Values assumed from the other probabilities.

**Table 3:** Results of cost-effectiveness assessment (deterministic analysis).

Treatment	Cost (BRL)	Incremental cost (BRL)	Effectiveness (QAPY)	Incremental Effectiveness (QAPY)	CER (BRL/QAPY)	ICER (BRL/QAPY)
CTP	579.16		5.17		16.11	
PTIS	2,949.55	2,370.40	10.27	5.11	52.96	464.22

CTP: conventional total prosthesis; ISTP: implant-supported total prosthesis; QAPY: quality-adjusted prosthesis year; CER: cost-effectiveness ratio; ICER: incremental cost-effectiveness ratio

## Main Assumptions of the Model

The objective of this analysis was to estimate the differences in costs and effectiveness of the two treatments, calculating their incremental cost-effectiveness ratios (ICER). To this end, the following assumptions were made:

1. Repeat treatment in case of therapy failure was not considered, because of the substantial uncertainty regarding its efficacy and adverse events.
2. Durability of the implant was set at 20 years (time horizon). Replacement time for both conventional and implant-supported prosthesis was assumed to be five years, considering textbook recommendations and the normal wear of the acrylic resin. This protocol is widely used in the SUS.
3. The total QAPY value was assigned to state A, for both treatments. For state B, a 25% decrease in QAPY was applied, and for state C, a 50% decrease. This approach was tested in the sensitivity analysis.
4. There was no established value to be used as reference for the threshold willingness to pay value of the evaluated treatments. Thus, we used the per-QALY value recommended by the World Health Organization (WHO)^19^. According to the WHO, a country’s per QALY spending should be between one and three times the value of its *per capita* gross domestic product (BRL 30,407.00 in Brazil). Thus, for a technology to be considered cost-effective, its cost may range from BRL 30,407.00 to BRL 91,221.00 per QALY. However, we infer that a QAPY should cost only a fraction of a QALY. Therefore, we assumed that it would be acceptable to pay up to 10% of the *per capita* GDP (BRL 3,050.00) per QAPY.

## Experts Panel

A panel of experts was organized to provide feedback on the parameters used in the model, so possible disagreements concerning incorporation of data and parametric assumptions could be identified. The panel was also tasked with assessing the quality of the available data. Questions were sent by e-mail to dental surgery and dental prosthesis researchers of different Brazilian universities.

These were open questions asking for information on the success rate of the treatments, clinical phases and abandonment rates, among others. As researchers, they were also suggested to send results of studies possibly able to contribute to the construction of the model. From the responses of five researchers, the model’s data and assumptions were confirmed.

## Model for Probabilistic Sensitivity Analysis

A Monte Carlo simulation with 1,000,000 iterations was performed in order to carry out probabilistic sensitivity analysis. Cost-effectiveness acceptability curves (CEACs) were presented as a decision-making approach, summarizing information on cost-effectiveness uncertainty. Analyzes were performed in Microsoft Excel^®^, using Visual Basic for Applications.

## RESULTS

Table 2 presents the costs of the treatments, including incremental cost (cost difference between the technology under analysis and the technology with lowest cost), effectiveness, incremental effectiveness and cost-effectiveness ratio (CER). The CER of each treatment is calculated by dividing its cost by its effectiveness. The ICER is calculated by dividing ISTP’s incremental cost by its incremental effectiveness; the result can then be compared to the reference strategy’s (CTP).

Compared to CTP, deterministic analysis showed that ISTP had an ICER of BRL 464.22 per QAPY. This is much lower than the threshold of 10% of GDP *per capita* (BRL 3,050.00) per QAPY assumed in the model, indicating that ISTP is a very cost-effective technology. The impact of uncertainty (in the adopted threshold and model parameters) over the results was evaluated in the sensitivity analysis.

### Probabilistic sensitivity analysis

**Figure 2:**
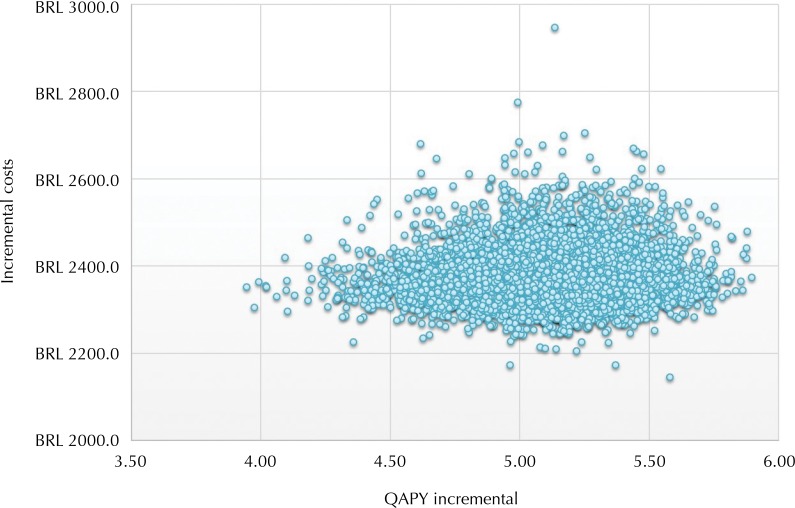
Incremental cost-effectiveness (implant-supported total prosthesis *versus* conventional total prosthesis).

Results of the Monte Carlo simulations are shown in Figure 2. Each circle represents a single simulation result, and shows the incremental effects and costs of ISTP in comparison to CTP.

QAPY: quality-adjusted prosthesis year

This analysis showed that rehabilitation treatment with ISTP offers an increase of effectiveness for the patient (as measured in QAPY), accompanied by an incremental cost for the health system, considering the specified time horizon.

### Cost-Effectiveness Acceptability Curve (CEAC)

**Figure 3:**
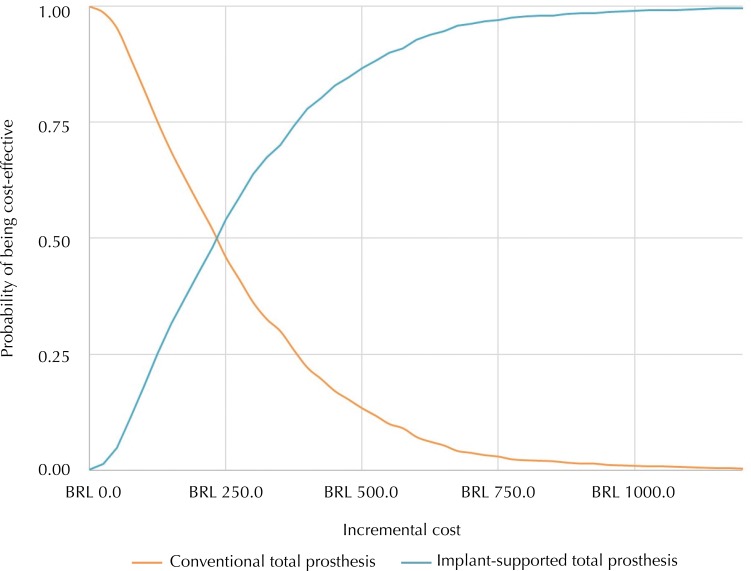
Acceptability curves of cost-effectiveness according to the applied time horizon (20 years).

CEAC results show that, over a time horizon of 20 years, the probability of ISTP being cost-effective is 54.15% for the BRL 250.00 threshold, 82.92% for the BRL 450.00 threshold, and 97.0% for the BRL 750.00 threshold (Figure 3). That is, the greater the willingness to invest in the technology, the more likely it is to be cost-effective. For the BRL 3,050.00 per QAPY willingness to pay threshold adopted in the model, ISTP had a 100% probability of being cost-effective.

## DISCUSSION

The results of this economic analysis showed that ISTP was cost-effective compared to CTP, considering the employed cost-effectiveness limits: that is, at a low incremental cost, the ISTP provides more effectiveness than CTP. This is in agreement with the differences in quality of life, adaptation, quality of mastication, and speech observed in other studies comparing the two rehabilitation techniques^9,29^.

Brazil has a human rights-based national oral health policy known as *Brasil Sorridente*^3^, which determines public actions for the promotion of oral health, as well as measures of prevention and recovery. In Australia, for example, public dental services (low-cost or fully subsidized) are only provided for people under the age of 18 and adults with health cards (issued by the Australian government to low-income earners and other selected groups). Chile also has a limited provision of public oral health services: in addition to basic outpatient emergency care, the country’s public health insurance is restricted to primary services for pregnant women, children up to six years of age, and adults over 60 years old^31^.

*Brasil Sorridente* proposes as one of its lines of action the expansion and qualification of specialized care, especially by establishing dental specialty centers and regional labs for dental prostheses^3^. There is also a provision for the oral rehabilitation of completely edentulous patients^11,12^. This reinforces the need for economic studies to be carried out in order to guide the rational and equitable allocation of available financial resources, maximizing the assisted population’s health gains.

The results of our evaluation should be interpreted in the light of certain limitations. It was challenging to accurately estimate rehabilitation costs for ISTP *versus* CTP. Our calculation estimate was based on the SIGTAP table^26^. Even though it is the SUS reference for health procedure prices, the table is not a good representation of oral health’s current reality. According to it, for example, consultations in primary care or even some secondary care procedures are unpaid. This limitation notwithstanding, the SIGTAP table was the only available source for the specific values the federal government passes on to the municipalities that perform the procedures evaluated here. It is also worth pointing out that, by adopting the Ministry of Health’s perspective, we did not take into consideration the expense municipalities have to front in order to offer the technology to its users. However, since the municipality’s perspective is also very relevant, we have already started a micro-accounting study to estimate precisely how much municipalities would have to spend, in an attempt to estimate whether Ministry of Health support is sufficient and, if otherwise, how much would be required to pay for the procedure’s inputs.

The QAPY values for total edentulous patients were calculated according to the proposal by Chun et al.^22^ The absence of studies estimating the QAPY of the completely edentulous population that has been rehabilitated by the evaluated technologies points to this study’s novelty as well as the lack of more in-depth dentistry research in the area of economic evaluations. However, it also suggests that the results presented here must be evaluated critically. Due to the multiple uncertainties regarding the parameters adopted in the model and in order to reduce them when presenting the results to the decision maker, we performed a probabilistic sensitivity analysis with one million iterations. Thus, we emphasize the need for studies that more solidly support full economic evaluations.

The lack of evidence in the literature leads us to another question: we were unable to find any reference for the per-QAPY willingness to pay threshold used in this study. As previously explained, this limitation was dealt with by assuming that it was reasonable to pay up to 10% of the national *per capita* GDP, according to a rationale based on the WHO-recommended per-QALY value. The determination of a willingness to pay threshold for a given technology is not simple and entails a number of issues, including the importance that the population attaches to the treatment in question, and whether the payment is sourced privately or from public health investment^14^. Although there is no research in Brazil evaluating the population’s willingness to pay for oral rehabilitation, a Canadian study showed that, under the hypothesis of becoming completely edentulous, individuals would be willing to pay a significant amount to receive total prostheses supported by two implants^32^. The Canadian study corroborates the rationale adopted here.

We emphasize that Brazil’s National Oral Health Survey (SB Brazil 2020) is currently in the planning stage. It would be appropriate for the issues discussed here to be contemplated by this national research. This would strongly contribute to a better planning of future health actions. SB Brazil 2020 may also provide important data for accurately calculating QAPY in mandibular edentulous people rehabilitated via ISTP or CTP.

The findings of this economic analysis cannot be generalized to all edentulous patients, since only mandibular edentulous patients were considered. However, they can be used to guide decision making when it comes to fulfilling the needs of those who require oral rehabilitation in the SUS. It is also important to point out that these results cannot be generalized to other countries, although the model itself can be reproduced in other scenarios.

During the development of this study, we observed a large amount of reports in the literature regarding the mandible’s difficulty of adaptation to CTP^19,20,22,33,34^. However, we did not find reports on the rate of abandonment of these prostheses or even the rate of failure of the implants in the SUS. Thus, we do not have concrete estimates on how many patients have received the treatment and ended up not using the conventional total prosthesis, or have undergone surgery and ended up having the implant biologically rejected. From the perspective of the SUS as a financer, both cases could be construed as a waste of resources.

The panel of experts informed us that, on average, 50% of patients abandon the lower CTP. If this information had been provided directly by the SUS, we would have been able to estimate how much is being spent to offer a technology that ends up being abandoned by the patient. These data would possibly show that the cost-effectiveness of ISTP is even better than anticipated, since costs with patients’ non-adaptation to CTP would also have been factored in. As there are already dental specialty centers in Brazil that offer the implant service for those who do not adapt to the conventional treatment, we intend to further pursue this line of research and carry out a primary study to obtain the data necessary to fulfill this gap.

The issue of implant technology cost-effectiveness is crucial for Brazilian dentistry researchers. When the technologies are compared in randomized clinical trials, the results are presented in an ideal context, failing to represent the reality of the various specialty centers in the country. Thus, the gathering of data on the effectiveness of these technologies, including the longitudinal monitoring of the performed treatments, would go a long way in overcoming the limitations encountered in this study’s development. We would also add that, although the experience of the *Brasil Sorridente* program can be considered unique, a look at this policy and its impacts on the population can serve as a reference to other countries when it comes to universal health systems’ defense of access to oral health.

We reinforce the importance of cost-effectiveness studies adopting the perspective of the SUS, especially when considering the budgetary challenges anticipated after the approval of Constitutional Amendment 95/2016^35^, establishing the country’s new fiscal regime. Based on the results found here, we conclude that the ISTP technique supported by two implants was a cost-effective means for the rehabilitation of completely mandibular edentulous patients in the SUS, as compared to CTP.
